# Circulating Endothelin 1 but Not Transforming Growth Factor-β Levels Are Reduced after Pulmonary Endarterectomy in Subjects Affected by Chronic Thromboembolic Pulmonary Hypertension: A Prospective Cohort Study

**DOI:** 10.3390/jcm13174977

**Published:** 2024-08-23

**Authors:** Pasquale Totaro, Claudio Tirelli, Mara De Amici, Fabrizio Grosjean, Giorgia Testa, Lucia Sacchi, Annalisa De Silvestri, Alessia Alloni, Eraldo Kushta, Riccardo Albertini, Teresa Rampino, Andrea Maria D’Armini

**Affiliations:** 1 Division of Cardiac Surgery 2,Pulmonary Hypertension Center, Foundation I.R.C.C.S. Policlinico San Matteo, 27100 Pavia, Italy; 2 Respiratory Unit, ASST Santi Paolo e Carlo, Department of Health Sciences, University of Milan, 20142 Milan, Italy; 3 Immuno-Allergology Laboratory of Clinical Chemistry, Foundation I.R.C.C.S. Policlinico San Matteo, 27100 Pavia, Italy; 4 Unit of Nephrology, Dialysis–Transplantation, Foundation I.R.C.C.S. Policlinico San Matteo, 27100 Pavia, Italy; 5 Pediatrics Clinic, Foundation I.R.C.C.S. Policlinico San Matteo, 27100 Pavia, Italy; 6 Department of Electrical, Computer and Biomedical Engineering, University of Pavia, 27100 Pavia, Italy; 7 Scientific Direction, Clinical Epidemiology & Biometric Unit, Foundation I.R.C.C.S. Policlinico San Matteo, 27100 Pavia, Italy

**Keywords:** endothelin 1, transforming growth factor-β, chronic thromboembolic pulmonary hypertension, pulmonary endarterectomy, mean pulmonary arterial pressure

## Abstract

**Background and objectives:** Endothelin-1 (ET-1) and transforming growth factor-β (TGF-β) play a pivotal role in the pathophysiology and vascular remodeling of chronic thromboembolic pulmonary hypertension (CTEPH) which is an under-diagnosed complication of acute pulmonary embolism (PE). Currently, pulmonary endarterectomy (PEA) is still the treatment of choice for selected patients suffering from CTEPH. The aim of this study was to evaluate the preoperative and postoperative circulating levels of ET-1 and TGF-β in subjects affected by CTEPH undergoing successful surgical treatment by PEA. **Methods:** The data from patients diagnosed with CTEPH who underwent PEA at the Foundation IRCCS Policlinico San Matteo Hospital (Pavia, Italy) were prospectively recorded in the Institutional database. Circulating ET-1 and TGF-β levels were assessed by an ELISA commercial kit before PEA, at 3 months and 1 year after PEA. The demographic data, preoperatory mean pulmonary arterial pressure (mPAP), cardiac output (CO), and pulmonary vascular resistance (PVR) were also recorded. Univariate and multivariate analyses were performed. **Results:** The analysis included 340 patients with complete ET-1 measurements and 206 patients with complete TGF-β measurements. ET-1 significantly decreased both at 3 months (*p* < 0.001) and at 1 year (*p* = 0.009) after PEA. On the other hand, preoperatory TGF-β levels did not significantly change after PEA. Furthermore, ET-1, but not TGF-β, was a good predictor for increased mPAP in multivariate analyses (*p* < 0.05). **Conclusions:** ET-1 but not TGF β was significantly modulated by PEA in subjects affected by CTEPH up to 1 year after surgery. The mechanisms leading to prolonged elevated circulating TGF-β levels and their clinical significance have to be further elucidated.

## 1. Introduction

Chronic thromboembolic pulmonary hypertension (CTEPH) is the most feared long-term complication after an acute pulmonary embolism (PE), and it is caused by incomplete fibrinolysis of thromboembolic material, evolving into fibrous plugs that obstruct the pulmonary arterial vasculature [1]. The incidence of CTEPH is estimated at 2% to 3% of all acute PEs and represents, therefore, an increasing and, so far, underestimated cause of hospitalization in the modern era [2].

CTEPH is the main condition associated with group IV pulmonary hypertension (PH).

PH is indeed classified into five clinical subgroups: pulmonary arterial hypertension (PAH) (group I), PH secondary to left-sided heart disease (group II), PH due to chronic lung disease (group III), chronic thromboembolic PH (CTEPH) (group IV), and PH with unclear and/or multifactorial mechanisms (group V) [3].

According to the guidelines, CTEPH diagnosis should be based on findings obtained after at least 3 months of anticoagulation. The diagnostic findings are as follows: (1) precapillary PH with a mean pulmonary artery pressure (mPAP) of ≥20 mmHg, with a pulmonary artery wedge pressure of ≤15 mmHg and a pulmonary vascular resistance (PVR) of >2 Wood units; (2) the detection of specific vascular lesions by multidetector computed tomography (CT) angiography, pulmonary cineangiography, or MRI such as chronic total occlusions or ringlike stenoses, causing segmental perfusion defects [3].

One of the main problems related to the correct diagnosis and definition of CTEPH is that it may also be present without prior venous thromboembolic history, and this could contribute to its under-recognition in the first instance. Symptoms of CTEPH, on the other hand, could be non-specific and very similar to those of other cardiopulmonary conditions. Therefore, a prompt diagnosis is surely a key issue, since a diagnostic delay negatively impacts CTEPH prognosis, with higher mortality risks.

The cornerstone of the successful treatment of patients suffering from CTEPH has been the introduction by Jamieson and Kapelansky, at the San Diego Medical Center, of a dedicated surgical treatment, the pulmonary endarterectomy (PEA) [4].

PEA surgery is a complex bilateral surgical procedure that requires sophisticated techniques and postoperative intensive care treatment. After a median sternotomy and cardiopulmonary bypass are performed, a gradual cooling (to 20 °C) is started while the arrest of the circulation is safely obtained [5]. During the deep hypothermic circulatory arrest intervals, the dissection and correct removal of thromboembolic material are performed [5].

Despite being reserved to a few centers with a high volume, PEA has become, in the last two decades, the treatment of choice for selected patients with CTEPH [4,5,6,7].

Endothelin-1 (ET-1) is a peptide composed of 21 amino acids encoded by the *EDN1* gene. It was identified as a potent vasoconstrictive metabolite [8].

ET-1 is released by endothelial cells. After its release, it has been demonstrated that ET-1 can cause potent and durable vasoconstriction through ET-A receptors located on vascular smooth muscle cells. Parallelly, when ET-1 binds to ET-B receptors on endothelial cells, it could induce vasodilation through the release of nitric oxide [9].

Given the vasoactive response induced by ET-1, its potential role in cardiovascular diseases and the effects of elevation in ET-1 circulating plasma levels have been studied and described in many vascular diseases, such as atherosclerosis, systemic hypertension, and pulmonary hypertension [10,11,12].

Further research has pointed out that ET-1 can act not only as a modulator of vasoreactivity but can also influence inflammation, myocardial contractility, and sodium excretion [13].

ET-1 is thus an endogenous peptide with a role in the pathophysiology of pulmonary arterial hypertension and other pulmonary diseases, such as chronic obstructive pulmonary disease (COPD) and bronchiectasis [14,15,16,17,18].

It has been demonstrated that elevated ET-1 is correlated with more severe hemodynamic impairment in patients with non-thromboembolic pulmonary hypertension; however, studies in CTEPH are limited [1,14,19]. It has also been shown that the ET-1/transforming growth factor-β (TGF-β) axis plays a pivotal role in vascular remodeling, leading to non-resolution of thrombus in CTEPH [20].

TGF-β is a pleiotropic factor conserved during evolution which can regulate lots of biological processes (immunity, development, tumorigenesis, and tissue regeneration). Moreover, TGF-β is crucial for epithelial–mesenchymal interactions during lung branching morphogenesis and alveolarization and in the epithelial–mesenchymal transition, leading to pulmonary fibrosis through fibroblast activation, extracellular matrix organization, and alveolar epithelial cell differentiation [21,22].

The role of ET-1 and TGF-β in vasculature remodeling and their relationship with PE are also under investigation in SARS-CoV-2-induced PE [23,24].

The aim of our study was to evaluate the hypothesis that, in subjects affected by CTEPH, the level of circulating ET-1 and TGF-β could be influenced by successful PEA, thus optimizing long-term postoperative outcomes.

## 2. Materials and Methods

We prospectively analyzed the data from three hundred and forty patients with CTEPH who underwent PEA at the Foundation IRCCS Policlinico San Matteo Hospital (a tertiary referring hospital for CTPEH, Pavia, Italy) over a 64-month period. All the patients received complete preoperative screening and signed an informed consent form for participation in the study.

Particularly, all the patients signed an informed consent form, approved by the Institutional Review Board of Foundation IRCCS Policlinico San Matteo (Pavia, Italy) for longitudinal, non-pharmacological, and non-sponsored studies, which complied with the Italian legislation (Codex on Privacy, D.Lgs 30 giugno 2003, n.196). The data were thus prospectively stored in a dedicated Institutional database. After the collection, the data were analyzed in this prospective, single-center, observational study. Demographic and hemodynamic data including preoperatory mean pulmonary arterial pressure (mPAP), cardiac output (CO), and pulmonary vascular resistance (PVR) at right heart catheterization were recorded for all the enrolled patients and are summarized in Table 1. Plasma ET-1 levels were measured, for all patients, by an enzyme immunoassay technique (Immunoassay Quantikine™ ELISA Catalog Number DET100, Bio-Techne R&D Systems, 614 McKinley Palace, NE Minneapolis, MN 55413 USA), according to the manufacturer’s instructions and expressed as pg/mL. In a further subgroup of 206 patients (46% males and 54% females), TGF-β serum levels were also measured by an enzyme immunoassay technique (ELISA Catalog Number DB100C, Bio-Techne R&D Systems 614 McKinley Palace, NE Minneapolis, MN 55413 USA), according to the manufacturer’s instructions and expressed as pg/mL. Both ET-1 and TGF-β levels were determined at baseline (during preoperative assessments before PEA) and postoperatively (during out-patient follow-up assessments) at 3 months and 1 year after PEA. The trends of ET-1 and TGF-β in postoperative outcomes were recorded and analyzed. The impact of pre- and postoperative ET-1 and TGF-β in postoperative outcomes was also analyzed.

## 3. Statistical Analysis

Statistical analysis was performed using the statistical software package Stata v17.0 (StataCorp, College Station, TX, USA). Descriptive statistics are expressed as medians and inter-quartile ranges. A non-parametric Friedman analysis of variance for repeated measures was used to evaluate changes in ET-1 and TGF-β over time. The correlations between plasma ET-1 values and plasma TGF-β levels were tested by Spearman’s correlation coefficient (r). Multivariable generalized equation estimation models were performed to predict mPAP based on sex, age, hemodynamic parameters, and quantiles of ET-1 and TGF-β. Only variables with *p* < 0.20 in univariate analyses were entered in the multivariable model. A *p*-value of 0.05 was considered statistically significant. The log-rank test for the Kaplan-Meier curve was used to evaluate the impact of ET-1 and TGF-β on long-term survival following PEA.

## 4. Results

### 4.1. Overall Operative Data

Twenty-five patients died in the hospital for an overall in-hospital mortality of 7.3%. Overall, the operative data and postoperative outcomes are summarized in Table 2. Extracorporeal circulation was mandatory in all procedures, with durations ranging from 115 to 721 min. Only a minority of patients (7%), on the other hand, required additional measures such as an aortic cross-clamp and cardioplegic diastolic cardiac arrest, mainly in the case of combined surgical myocardial revascularization. A further 12% of patients required combined procedures, however, without the need for cardioplegic cardiac arrest. All the procedures were conducted in mild–moderate hypothermia, and, in order to facilitate surgical accuracy, a number of short (5–7 min) intervals of cardiocirculatory arrest (ranging from 2 to 26) were also adopted with an overall cardiocirculatory arrest time ranging from 11 to 192 min. Early postoperative extracorporeal membrane oxygenation (ECMO) to facilitate patient weaning or to address significant respiratory bleeding was necessary in 4% of patients. Among the postoperative complications, of note, 26.4% of patients developed arrhythmic events, and 7.3% needed a tracheostomy, while 8.2% had neurological sequelae. Of interest, the mean in-hospital stay was 16 days, and the mean intensive care unit (ICU) stay was 8 days.

### 4.2. Correlations between Preoperative ET-1 and TGF-β Levels and Hemodynamic and Clinical Parameters

Preoperative plasma ET-1 levels significantly correlated with mPAP (Spearman coefficient r = 0.12, *p* = 0.015), whereas plasma TGF-β levels did not, despite the significant correlation found between preoperative plasma ET-1 and TGF-β (r = 0.21, *p* = 0.0011). Furthermore, in the multivariate analysis (Table 3), preoperative ET-1 resulted in being the only good predictor for increased mPAP. Preoperatory ET-1 levels were, indeed, higher in patients with preoperative mPAP > 40 mmHg while no difference was seen for TGF-β (Table 4).

**Figure 1:**
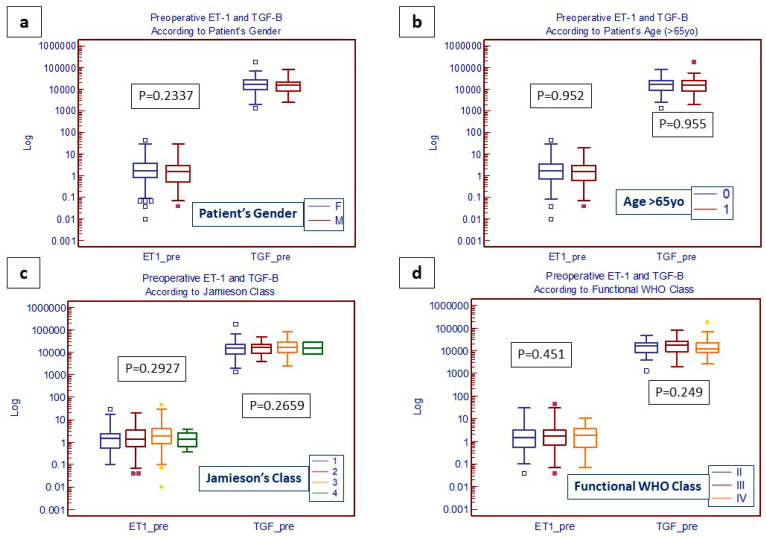
Box and whiskers plot of preoperative ET-1 (A) and TGF-β (B) levels according to patient demographic characteristics (**a**,**b**); extension of diseases (**c**); and clinical status (**d**).

When considering a stratification of the patients according to preoperative symptoms (Table 5), the results showed a statistically significant correlation between ET-1 levels and both mPAP and pulmonary vascular resistance (PVR) but only in patients presenting with limited symptoms (WHO class II). On the contrary, no significant correlations were observed between TGF-β and the hemodynamic parameters regardless of the severity of preoperative symptoms. A history of the previous episode of deep venous thromboembolism (DVT) did not influence the preoperative values of either ET-1 (*p* = 0.3397) or TGF-β (*p* = 0.0748). Neither ET-1 nor TGF-β preoperative levels were influenced by the patient’s sex, age, level of disease extension (Jamieson class), or severity of clinical symptoms (functional class WHO), as shown in Figure 1.

### 4.3. Evaluation of Post-PEA Levels of ET-1 and TGF-β

**Figure 2:**
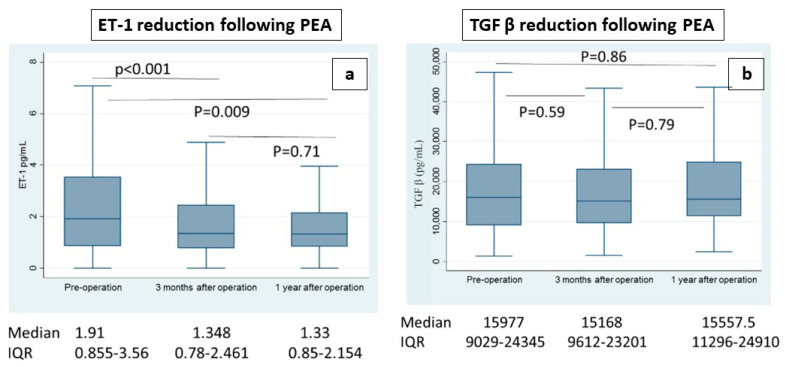
Box and whiskers plot of ET-1 (**a**) or TGF-β (**b**) levels in preoperative samples, at 3 months and 1 year after PEA.

PEA did significantly impact plasma ET-1 levels (Figure 2a) which were, indeed, significantly lower at both 3 months (*p* < 0.001) and 1 year after surgery (*p* = 0.009) compared to the baseline levels. No significant difference in ET-1 levels was retrieved between 3 months and 1 year after the operation. Furthermore, conversely to what was shown for the preoperative levels, the postoperative ET-1 levels were not influenced by the preoperative degree of PH (Table 4).

On the other hand, PEA did not seem to impact plasma TGF-β levels (Figure 2b) as they did not change significantly in the postoperative course either at 3 months or 1 year. TGF-β levels, furthermore, seemed to not be correlated with the preoperative degree of PH at any stage of evaluation either pre- or post-PEA (Table 3).

**Figure 3:**
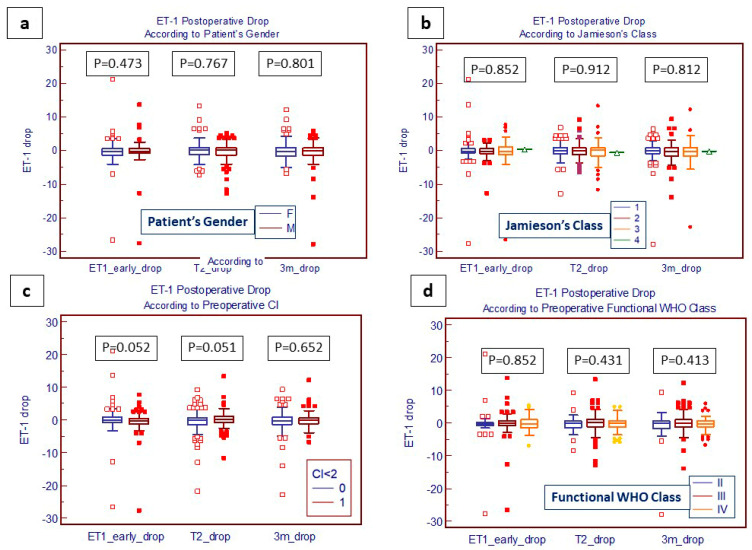
Box and whiskers plot of ET-1 postoperative drop according to patient’s gender (**a**), extension of disease (**b**), and hemodynamic and clinical parameters (**c**,**d**). ET1 early drop: ET-1 drop at hospital discharge; T2 drop: ET-1 value drop from discharge to 3-month follow-up; 3 m drop: ET-1 value cumulative drop from preoperative to 3-month follow-up.

Early and 3-month ET-1 drops were not significantly influenced neither by the patient’s gender nor by the preoperative extension of diseases according to Jamieson’s classifications, and nor by the severity of preoperative symptoms defined according to the clinical functional status WHO classes and hemodynamic dysfunction (Figure 3a–d). Only a preoperative Cardiac Index < 2 seems to be at the limit for statistical significance in influencing the postoperative early and 3-month drop of ET-1 levels.

### 4.4. Impact of ET-1 and TGF-β Levels on Long-Term Outcomes Following PEA

Finally, we tried to correlate the preoperative increased levels of ET-1 and TGF-*β* with long-term postoperative outcomes following PEA.

**Figure 4:**
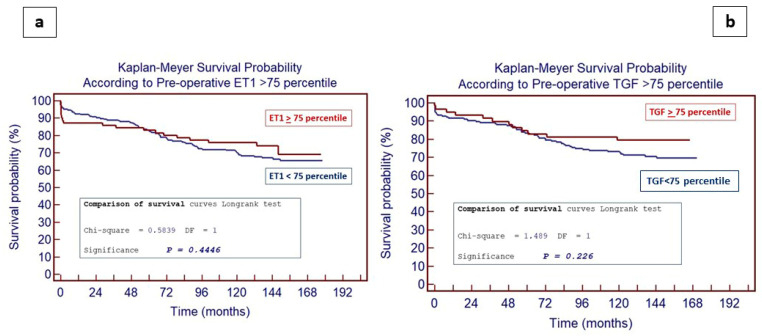
Long-term (>15 years) survival following PEA according to preoperative value of plasma ET-1 (**a**) and serum TGF-β (**b**).

**Figure 5:**
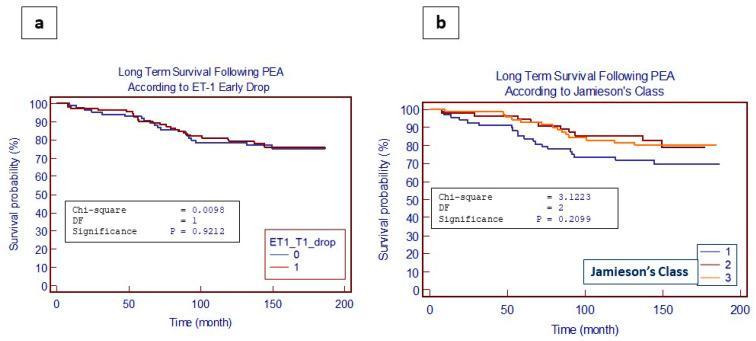
Long-term (>15 years) survival following PEA according to ET-1 postoperative drop at 3 months (**a**) and preoperative extent of disease (**b**).

A total of 315 patients were discharged from the hospital (205 for the TGF-β subgroup) and entered a follow-up (from 0 to 185 months). We evaluated the impact of the significantly increased (defined as value >75 percentile) preoperative ET-1 (Figure 4a) and TGF-β (Figure 4b), and no influence on long-term mortality following PEA was shown. Long-term survival, furthermore, was not influenced neither by the preoperative extension of disease (according to Jamieson’s classification) nor by the ET-1 drop at 3 months, as shown in Figure 5a,b.

## 5. Discussion

Chronic thromboembolic pulmonary hypertension (CTEPH) is the most feared long-term complication after an acute pulmonary embolism (PE), accounting for up to 2–3% of all acute PE [25]. PEA is considered the best treatment option for technically operable patients. Notably, more recently, another technique has been introduced, namely balloon pulmonary angioplasty (BPA), which allows for the percutaneous revascularization of pulmonary vascular obstructions for patients who are not eligible for PEA or have residual signs of obstruction after PEA [26].

Growing interest has emerged in recent years for the metabolomic and biological profiles of CTEPH, particularly in how specific metabolites and potential biomarkers can be affected by PEA in CTEPH patients.

In the present study, we analyzed the variation in plasma levels of ET-1 and TGF-β in a large population of patients affected by CTEPH who underwent PEA. Although this was a single-center study, it represents to the best of our knowledge the first one including such a large number of patients (340) which prospectively focuses on the variation in plasma levels of ET-1 and TGF-β after PEA.

Our findings confirmed that PEA was a safe procedure, although, also in our series, an in-hospital mortality rate of 7.3% was registered in more frail or comorbid patients, in accordance with data from the literature. Moreover, in our cohort, ECMO was needed in just 4% of cases, a lower rate compared to data from series in the literature of about 9% [27].

In our cohort, preoperative plasma ET-1 levels significantly correlated with mPAP, whereas plasma TGF-β levels did not. These results might be interpreted considering the vasoconstrictive effect ET-1 plays in the vasculature milieu, which has a recognized and relevant role in pulmonary hypertension since antagonists of its receptor are currently adopted in the pharmacotherapy of idiopathic pulmonary hypertension.

Moreover, DVT, one of the main risk factors of PE, did not affect neither ET-1 nor TGF-β levels.

Notably, we found that, in a large population of patients affected by CTEPH and treated with PEA, plasma ET-1 levels decreased significantly following successful surgical treatment. Moreover, preoperatory ET-1 values correlate with the follow-up ones. Similar results were observed by other groups in studies, including a limited number of patients undergoing both PEA [14] and balloon pulmonary angioplasty [28,29], the previously cited less invasive treatment that was recently introduced as an alternative or in conjunction with a previous PEA.

We also found that both early ET-1 level drops after PEA and at 3 months after surgery were not significantly influenced by the patient’s gender, the preoperative extension of diseases according to Jamieson’s classifications, or the severity of preoperative symptoms as defined according to the clinical functional status WHO classes and hemodynamic dysfunction. These data are noteworthy, since, once again, we confirmed the role PEA played in the reduction in ET-1 levels independently from potential confounders or co-contributing factors.

Our data are surely peculiar as we were able to confirm, on the one hand, the tight correlation, in patients suffering from CTEPH, between the preoperative values of plasma ET-1 levels and the severity of pulmonary hypertension [6], and, on the other hand, we were also able to show an original finding that the TGF-β production seemed to not be directly associated with the increased intravascular pressure. These data were both confirmed using multivariate analyses, where ET-1, but not TGF-β, resulted in being a good predictor for mPAP.

Furthermore, our data pointed out a novel feature regarding the preoperative interaction between ET-1 and TGF-β in patients with CTEPH awaiting to be treated with PEA.

It has been previously shown that TGF-β plays a major role in vascular remodeling and matrix deposition in pulmonary hypertension [1,30]. Accordingly, with previous in vitro studies showing that TGF-β induces ET-1 production in pulmonary arterial smooth muscle cells (PASMCs) [31] and endothelial cells [32], we have found a significant correlation between preoperative plasma ET-1 and TGF-β levels in the analyzed population of patients with CTEPH awaiting surgical treatment. Previous in vitro studies demonstrated that ET-1 and TGF-β act synergistically, inducing PASMC proliferation, endothelial-to-mesenchymal transition, and extracellular matrix deposition [33,34]. Furthermore, it has been demonstrated that endothelin receptor blockers reverse TGF-β signaling overactivation and that ET-1 expression is increased in chronic murine venous thrombi, human CTEPH endothelial cells, or PEA specimens [20]. Interestingly, the second finding of our study was that TGF-β and ET-1 levels were no longer correlated after a successful PEA. PEA was indeed associated with a significant decrease in ET-1 levels just after 3 months from surgery; on the contrary, TGF-β levels remained unchanged and seemed to not be influenced by a successful PEA.

Of note, the recurrence of CTEPH after PEA is very rare, although persistent exercise limitation following successful pulmonary endarterectomy has also been reported [35,36]. Since we showed that ET-1 circulating levels are reduced after PEA, it can be speculated that the surgical removal of occluding material from the pulmonary artery might eliminate either ET-1 production factors or ET-1 production sites. Nevertheless, our data seem to suggest that ET-1 plays a crucial role in vascular remodeling in CTEPH rather than circulating TGF-β alone. On the other hand, recent reports on the correlation between TGF-β-induced protein and impaired venous thrombus resolution deserve careful evaluation [37].

A few other findings of our study deserve further comment. Regarding the potential correlation between the preoperative levels of ET-1 and TGF-β with clinical symptoms of patients with CTEPH awaiting surgical correction, it was curious to notice the lack of correlation between the preoperative levels of both ET-1 and TGF-β and the hemodynamic parameters evaluating the degree of severity of pulmonary hypertension in patients presenting with severe functional limitations (WHO class III and IV). We could speculate that the impact of ET-1 circulating levels on the remodeling of intravascular material is especially relevant in the initial phase of CTEPH development and, therefore, easier to confirm in patients with a recent onset of clinical symptoms.

Finally, in our study, a correlation between the preoperative increased levels of ET-1 and TGF-β with long-term postoperative outcomes following PEA was conducted, considering the impact of significantly increased (defined as value > 75 percentile) preoperative ET-1 and TGF-β levels. Curiously, no influence on long-term mortality following PEA was detectable. Moreover, long-term survival did not seem to be influenced by the preoperative extension of the disease (according to Jamieson’s classification). These data confirmed once more that revascularization after PEA can improve the hemodynamics and that the surgical act is an independent factor of prognosis improvement, which is independent both by the preoperative extension of disease and the level of ET-1 and TGF-β.

Although our results are supported by a wide and adequate sample and data were prospectively collected, one limitation of the study is its single-center design. Further multicenter studies are needed to confirm our results and to elucidate the mechanism leading to prolonged elevated circulating TGF-β levels after PEA. On the other hand, the clinical implication of both the abnormal preoperative values of ET-1 and TGF-β as well as the postoperative response to the PEA needs to be completely evaluated, especially in terms of real impacts on long-term postoperative outcomes.

As far as our knowledge, however, our study is the first to clearly show that, despite being strictly correlated to the degree of pulmonary hypertension, the preoperative value of ET-1 is not correlated to a worse long-term outcome following a successful pulmonary endarterectomy.

## 6. Conclusions

In our study, we showed that, in patients with CTEPH treated with PEA, the ET-1 circulating level decreases significantly early after PEA and remains significantly lower for up to 1 year postoperatively. On the other hand, TGF-β production remains dysregulated for up to 1 year despite successful PEA. Long-term ET-1 and TGF-β circulating changes and their clinical significance, however, have to be further clarified with larger multicenter prospective studies.

## Figures and Tables

**Table 1:** Preoperative patient characteristics: BSA = body surface area; DVT = deep venous thrombosis; COPD = chronic obstructive pulmonary disease; MAP = mean arterial pulmonary pressure; CO = cardiac output, PVR = pulmonary vascular resistance; PAD = peripheral artery disease; CRF = chronic renal failure; and AF = atrial fibrillation.

Overall Patients Enrolled n.340			
**Gender**		**mPAP** **(mmHg)**	
** Male**	**145 (42.6%)**	** Range**	**12–81**
** Female**	**195 (57.3%)**	** Mean ± sd**	**44 ± 12**
**Age (Years)**		**CO (L/min)**	
** Range**	**18–83**	** Range**	**1.60–8.50**
** Mean ± sd**	**61 ± 14**	** Mean ± sd**	**3.90 ± 1.81**
**Smoking History**		**PVR (dyn·s/cm^5^)**	
** Yes**	**26 (7.6%)**	**PVR [Wood units]**	**78–2044 ** **[0.975–25.55]**
** No**	**204 (60%)**	** Range**	**851 ± 379**
** Previous**	**107 (31.4%)**	** Mean ± sd**	**[10.63 ± 4.73]**
		**WHO Class**	
**Weight (kg)**	**73 ± 17**	** II**	**56 (16.5%)**
**Height (m)**	**1.66 ± 10**	** III**	**175 (51.5%)**
**BSA (m^2^)**	**1.82 ± 0.24**	** IV**	**109 (32%)**
**History of DVT**	**181 (53.2%)**	**Hypertension**	**126 (37%)**
**O_2_ Therapy**	**158 (46.4%)**	**PAD**	**6 (1.7%)**
**COPD**	**54 (15.8%)**	**CRF**	**23 (6.7%)**
**Diabetes**	**17 (5%)**	**AF**	**7 (2%)**

**Table 2:** Intra-operative and postoperative patient characteristics: ECC: extracorporeal circulatory circulation, NF: nasopharyngeal; CA: circulatory arrest; ICU: intensive care unit; and ECMO: extracorporeal membrane oxygenation.

Intraoperative Parameters		Postoperative Parameters	
ECC Time (min)		In-Hospital Mortality	25 (7.3%)
** ** Range	115–721		
** ** Mean ± sd	343 ± 77		
Cross-Clamp Time (pts)	23 (7%)	ICU Stay (day)	
** ** Range (min)	17–86	** ** Range	0–83
** ** Mean ± sd (min)	33 ± 17	** ** Mean ± sd	8 ± 13
NF Temperature (°C)		In-Hospital Stay (day)	
** ** Range	21–30	** ** Range	0–100
** ** Mean ± sd	24 ± 1	** ** Mean ± sd	16 ± 12
CA (number of events)		Postoperative Complications	
** ** Range Mean ± sd	2–26 12 ± 4	** ** Arrhythmia ** ** ECMO ** ** Tracheostomy ** ** Neurological	90 (26.4%) 14 (4.1%) 25 (7.3%) 28 (8.2%)
CA Total Time (min)		Caval Filter	298 (87.6%)
** ** Range	11–192		
** ** Mean ± sd	89 ± 32		
Combined Procedure	64 (19%)		

**Table 3:** Multiple regression analysis. Multivariable generalized equation estimation model with mPAP as the dependent variable and sex, age, hemodynamic parameters, and the quantiles of ET-1 and TGF-β as predictors. Only variables with *p* < 0.20 at univariate analyses were entered in the model. TGF-β: transforming growth factor β; ET-1: endothelin 1; and 95% CI: 95% confidence interval.

	Coefficient	*p*-Value	95% CI
ET-1	0.37	***<0.001***	0.21–0.53
TGF-β	−0.05	0.54	−0.21–0.11
Sex	−0.04	0.936	−0.98–0.91

**Table 4:** Median (95% CI) of ET-1 or TGF-β levels in preoperative samples, at 3 months and 1 year after PEA according to preoperative mPAP. TGF-β: transforming growth factor β; ET-1: endothelin1; mo: months; y: year; PEA: pulmonary endarterectomy; and mPAP: mean pulmonary arterial pressure.

**Value**	**mPAP ≤ 40** **(n.125)**	**mPAP > 40** ** (n.215)**	***p***
ET-1 pre	1.35 (1.00–1.72)	1.87 (1.54–2.24)	***0.0174***
ET-1 3 months	1.22 (0.93–1.59)	1.29 (1.07–1.69)	0.5245
ET-1 1 year	1.40 (1.08–1.83)	1.28 (1.06–1.52)	0.5084
**Value**	**mPAP ≤ 40 ** ** (n.80)**	**mPAP > 40 ** ** (n.126)**	***p***
TGF-β pre	17,794 (14,408–19,536)	14,679 (11,061–17,398)	0.2422
TGF-β 3 months	16,182 (13,409–18,499)	15,065 (12,958–18,269)	0.8212
TGF-β 1 year	15,736 (13,951–17,721)	15,529 (13,500–17,394)	0.7824

**Table 5:** Spearman rho and *p*-value between preoperative ET-1 and TGF-β levels and hemodynamic parameters stratified according to WHO classification. CO: cardiac output; PVR: pulmonary vascular resistance; TGF-β: transforming growth factor β; ET-1: endothelin 1; and mPAP: mean pulmonary arterial pressure.

**ET-1**	**Class II (n = 56)**		**Class III (n = 175)**		**Class IV (n = 109)**	
	**r**	***p***	**r**	***p***	**r**	***p***
**mPAP**	***0.286***	***0.016***	0.052	0.47	0.139	0.12
**CO**	−0.072	0.55	0.005	0.94	0.070	0.44
**PVR**	***0.236***	***0.048***	0.013	0.85	−0.010	0.91
**TFG-** **β**	**Class II (n = 44)**		**Class III (n = 103)**		**Class IV (n = 59)**	
	**r**	***p***	**r**	***p***	**r**	***p***
**mPAP**	0.179	0.2298	0.160	0.0880	0.130	0.2775
**CO**	−0.0085	0.5668	0.0066	0.4824	0.034	0.7765
**PVR**	0.197	0.1857	0.074	0.4288	−0.121	0.3117

## Data Availability

The data presented in this study are available upon request from the corresponding author.
